# Parameterization of Fuel-Optimal Synchronous Approach Trajectories to Tumbling Targets

**DOI:** 10.3389/frobt.2018.00033

**Published:** 2018-04-05

**Authors:** David Charles Sternberg, David Miller

**Affiliations:** Space Systems Laboratory, Department of Aeronautics and Astronautics, Massachusetts Institute of Technology, Cambridge, MA, United States

**Keywords:** trajectory, parameterization, synchronous, docking, fuel minimizing

## Abstract

Docking with potentially tumbling Targets is a common element of many mission architectures, including on-orbit servicing and active debris removal. This paper studies synchronized docking trajectories as a way to ensure the Chaser satellite remains on the docking axis of the tumbling Target, thereby reducing collision risks and enabling persistent onboard sensing of the docking location. Chaser satellites have limited computational power available to them and the time allowed for the determination of a fuel optimal trajectory may be limited. Consequently, parameterized trajectories that approximate the fuel optimal trajectory while following synchronous approaches may be used to provide a computationally efficient means of determining near optimal trajectories to a tumbling Target. This paper presents a method of balancing the computation cost with the added fuel expenditure required for parameterization, including the selection of a parameterization scheme, the number of parameters in the parameterization, and a means of incorporating the dynamics of a tumbling satellite into the parameterization process. Comparisons of the parameterized trajectories are made with the fuel optimal trajectory, which is computed through the numerical propagation of Euler’s equations. Additionally, various tumble types are considered to demonstrate the efficacy of the presented computation scheme. With this parameterized trajectory determination method, Chaser satellites may perform terminal approach and docking maneuvers with both fuel and computational efficiency.

## Introduction

On-orbit servicing and active debris removal share many common characteristics. Such missions require at least two objects to interact in proximity: the Chaser satellite and Target object. There are currently orbiting Targets for both mission architectures that may be taken to be uncooperative and naturally tumbling, while requiring soft docking to prevent damage to the Chaser or Target. The necessity to perform soft dockings requires the Chaser to expend fuel, so approach trajectories that minimize fuel use are desirable. Additionally, the Chaser must be able to generate its approach trajectory without extensive computation to stay within computational constraints imposed by the Chaser’s hardware or to adapt to changes in the environment. Therefore, this paper addresses the need for a computationally efficient means of generating fuel efficient trajectories that can enable safe dockings to potentially tumbling Targets.

Autonomous Chasers are necessary, since the docking phase of servicing and debris removal missions occurs at the Target’s location. During the far rendezvous, ground-based control can adequately maneuver the Chaser satellite into position. The final approach and soft docking to the Target satellite, however, occur at time scales that prevent ground-based control: autonomous systems are required to perform the complex motions for soft docking. This time scale poses an urgency for the Chaser to compute its trajectory while avoiding excessive fuel consumption, especially when driven by a tumbling Target (NASA Goddard Space Flight Center, 2010). Autonomous systems can also be used to explore new areas of the servicing or debris removal tradespace, especially for locations beyond Low Earth Orbit and of Targets of multiple types and tumbles, without imposing a risk to humans (NASA Goddard Space Flight Center, 2010). Furthermore, autonomous Chasers can reduce mission costs by decreasing the required ground and communications infrastructure required (Gurevich and Wertz, 2001; Wertz, 2003). The benefits of autonomous operation justify designing Chaser satellites to be capable of autonomous trajectory generation and control.

One of the more important tasks of the autonomous Chaser is that of trajectory generation for the terminal approach for docking. Methods for generating safe, optimal trajectories are the focus of ongoing research. Planning information for these trajectories may be acquired from one or more onboard sensors (Sternberg et al., 2015), requiring estimation frameworks like that presented by Setterfield et al. (2017). There have been many optimization cost functions for generating approach trajectories. Engineers may define trajectory optimality for each mission scenario, but typically optimization cost functions include elements for fuel, time, and thruster activity (Robertson, 1968; Jackson, 1994; Paluszek and Thomas, 2005; Miele et al., 2007a,b; Bevilacqua et al., 2009; Vazquez et al., 2011; DiGirolamo et al., 2014). Constraints may also be added to the optimization process. These constraints often are imposed to ensure that Chaser satellite can operate safely. For example, to ensure that thruster plumes do not impinge on the Target satellite, Mixed-Integer Linear Programs have been studied by Richards et al. (2002) and Henshaw and Sanner (2010). Safety-based trajectories are assessed by Breger and How (2008). An assessment of the safety of different trajectories was studied in Luo et al. (2011). Therefore, there exist optimization frameworks for safe, fuel efficient docking trajectories.

Because the optimization process may require extensive computation time, however, research efforts have focused on creating rapid trajectory generation techniques. The resulting trajectories may be for the satellites themselves (McInnes, 2000; Munoz, 2011; Munoz and Fitz-Coy, 2013), or for manipulators attached to the Chaser satellite (Lampariello and Hirzinger, 2013). Combining the trajectory planning for the Chaser and its manipulators has also been studied (Rekleitis et al., 2007). Constraints have also been incorporated into these techniques. For example, nonlinear optimization for avoiding collisions between the Chaser and Target has been studied by treating the problem of collision avoidance as a nonlinear boundary value problem (Stoneman and Lampariello, 2016). This approach, while ensuring that the Chaser satellite will not hit a tumbling Target at any point during the rendezvous, is computationally efficient for the Chaser, but requires prior computation on the ground. The planning method is shown to find a global optimum for the defined problem in 50 min, requiring a ground-based component prior to online execution of a reference-tracking controller. These examples show that there is a continued need for trajectory optimization schemes that combine fuel and computational efficiency.

This paper considers the need for balancing computational efficiency with the fuel cost associated with the approximation of the fuel optimal trajectory for the scenario of a Chaser satellite conducting an approach and docking maneuver with a Target satellite. Additionally, it is based on the work presented in the doctoral thesis by the first author (Sternberg, 2017), and it assumes that the docking falls within the feasibility space for the Chaser to dock with the Target (Sternberg and Miller, 2017). The Chaser satellite is taken to have accurate state estimation and control. The Target satellite is assumed to be uncommunicative, rigid, without flexible structures, or fuel slosh. Additionally, the Target is assumed to be passive and not actively thrusting or otherwise creating motions or disturbances to its natural tumble. The Target may be tumbling, spinning, or nutating, and the docking axis may not be aligned with either the spin, body, or principle moment of inertia axes. To minimize the potential for undesired contact between the Chaser and Target, and to maintain sensor lock on the Target, the Chaser is constrained to remain synchronous with the Target. This synchronicity enforces that the Chaser approaches along the docking port axis of the Target. The short duration of the approaches allows the relative orbital dynamics to be excluded (the approaches are on the order of a tenth the orbital period or less), and the short, synchronous approach allows the Chaser to approach the Target along a rotating radial direction that decreases the likelihood of a collision between the Chaser and Target. Docking with tumbling targets have also been studied in the past, but without the synchronous approach as their focus (Boyarko et al., 2011; Virgili-Llop et al., 2017; Ventura et al., 2017; Wilde et al., 2017). Additionally, these other sources do not minimize the computational time required to obtain the fuel optimal trajectory through the parameterization of the approach trajectory to a set of basis functions of a reduced order. This paper first describes the method by which the Target’s tumble proscribes the motion of the Chaser along a synchronous path. To study fuel optimality, this paper uses the net Δ*V* as a metric for the required Chaser fuel consumption. A method of finding the fuel optimal trajectory is presented for a specified docking time. Several parameterization schemes are analyzed to reduce the computational complexity of generating the fuel optimal trajectory, and a reduced parameterization is studied for multiple tumbles. This paper identifies that two-term exponential expressions are able to provide a computationally efficient means of generating fuel efficient approach trajectories for several potential Target tumbles. Results are shown, for examples, drawn from potential servicing and debris removal scenarios, but the approach and key findings can be applied to support the development of an infrastructure for these and other mission architectures.

## Modeling Satellite Dynamics

**Figure 1:**
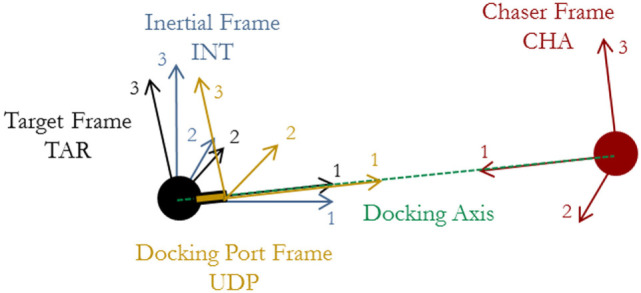
Definition of reference frames.

In order to model the Chaser’s approach, the behavior of the Target must be simulated as it tumbles. Several reference frames are used to describe the motion of both the Target and Chaser: those of the Target (TAR), docking port (UDP), inertial (INT), and Chaser (CHA). These reference frames are shown in Figure 1. Variables have superscripts to define the frame in which the quantity is measured, and for instances where both subscripts and superscripts are used, the quantity is a relationship from the subscript to the superscript (such as a quaternion from one axis system to another). The INT frame shares the same origin as the TAR frame, though TAR is body-fixed to the Target. The Chaser maintains synchronicity along its approach by keeping its axis system aligned along the docking axis. The UDP frame defines the docking axis, and it is translated by *r_f_* along the body axis of the Target.

The frames allow several rotations to be defined between them. To describe the transformation from one frame to another, quaternions are used: $q_{Y}^{X}$ is the quaternion from the *Y* frame to the *X* frame. For example, $q_{\text{INT}}^{\text{CHA}}$ is the quaternion from the inertial frame to the Chaser’s body frame, assuming that the Chaser’s docking port frame is co-aligned with the Chaser body frame.

The behavior of the Chaser and Target satellites is modeled by the dynamics is shown in Eq. 1 through Eq. 6. These equations describe the velocity profile that the Chaser satellite must follow if it is to remain along the docking axis of the Target, which is taken to be rotating at ω^TAR^. The quaternion multiplications in Eqs 1 and 2 represents the need for the two satellites to be oriented such that the docking ports are aligned and capable of docking: quaternion multiplication with $q^{Z_{\text{rot}}}$ rotates the orientation of the Target’s docking port in the inertial frame about the vertical axis to obtain the quaternion the Chaser must attain for both docking ports to align

(1) $$q_{\text{INT}}^{\text{UDP}}=q_{\text{TAR}}^{\text{UDP}}⊗q_{\text{INT}}^{\text{TAR}}$$

(2) $$q_{\text{INT}}^{\text{CHA}}=q_{\text{INT}}^{\text{UDP}}⊗q^{Z_{\text{rot}}}.$$

Equations 3–6 are used to compute the resulting velocity that the Chaser must attain. These equations do not have a specific computation of the rotation rate of the Chaser, since both Chaser and Target must rotate at the same rate to maintain alignment. Equation 3 represents Euler’s equations, which are propagated over a series of time steps to determine the angular acceleration of the target resulting from its inertia, moment, and net torques

(3) $${\dot{\omega }}^{\text{TAR}}=I^{{\text{TAR}}^{-1}}\left(-\omega^{\text{TAR}}\times \left(I^{\text{TAR}}\omega^{\text{TAR}}+M^{\text{TAR}}\right)+\tau^{\text{TAR}}\right).$$

Equation 4 determines the rate of change of the Target’s quaternion resulting from its tumble. Here, *M*^TAR^ is the Target’s internal momentum, and τ^TAR^ is the applied torque in the Target’s frame (which could include external disturbance torques). This equation, therefore, allows the orientation of the Target to be computed at each time step by the quaternion rate of change equation

(4) $${\dot{q}}_{\text{INT}}^{\text{TAR}}=\frac{1}{2}\left({\left[\omega^{{\text{TAR}}^{\text{T}}};0\right]}^{\text{T}}\right)⊗q_{\text{INT}}^{\text{TAR}}.$$

The velocity of the chaser is computed by integrating the angular acceleration of the Target’s tumble and taking the cross product between the angular rate and the vector from the Target to the Chaser satellite

(5) $$\omega^{\text{TAR}}=\int_{0}^{t_{\text{end}}}{\dot{\omega }}^{\text{TAR}}\mathrm{dt}$$

(6) $${\text{ν}}^{\text{CHA}}=\omega^{\text{TAR}}\times r_{\text{TAR}}^{\text{CHA}}.$$

In this equation, $r_{\text{TAR}}^{\text{CHA}}$ is the vector from the Target to the Chaser. These equations are, therefore, based on the properties of the Target satellite’s dynamic state and physical parameters. Importantly, these sets of dynamics do not account for relative orbital dynamics, since the terminal approaches are taken to occur over a sufficiently short duration as compared to the Target’s orbital period. The computation of the velocity of the Chaser satellite at each time step enables both the propagation of the Chaser’s position with time and the determination of the change in velocity at each time step necessary to maintain synchronicity. Therefore, these equations set up the computation of the Chaser’s fuel requirements.

## Computing Fuel Costs

In order to determine fuel optimal trajectories, a process must be created to compute the fuel requirements for various tumble types. This process is designed to be generic and based on the dynamics of the Chaser’s approach to a tumbling Target so that any trajectory may be analyzed. Throughout the approach, the Chaser satellite will be subject to the accelerations both to maintain synchronicity with the Target’s docking axis as well as to close the distance from its initial position to the final docking radius. For this work, the Target’s docking port is taken to be a distance *r_f_* from the center of rotation of the Target and that the Chaser’s approach begins a distance *r_0_* from the Target’s center of rotation along the docking axis. Furthermore, the Chaser is taken to be moving along the docking axis of the Target. With this set of initial conditions, the Chaser begins its approach by adjusting its trajectory from one that is orthogonal to the docking axis to an accelerated trajectory inward along the docking axis.

The constraint of maintaining synchronicity throughout the terminal approach to the rotating Target forces the Chaser to follow an accelerated profile, i.e., the Chaser must use its thrusters throughout the maneuver. For this analysis, the Chaser is assumed to have the ability to fire its thrusters at any magnitude as required. This assumption enables the assessment of Chaser satellite requirements without placing constraints on the satellite’s design. The assumption was made to allow further analysis into the feasibility of multiple families of trajectories. The inclusion of minimum or maximum firing times representative of a pulse-width modulated thruster system would increase the fuel use away from the obtained results because of the discretization of the required thruster firing, though only small modifications to the presented approach would be necessary to account for this Chaser satellite-specific characteristic. Additionally, the Chaser is assumed to have a controller which will enable it to follow the desired trajectory. This reference-tracking controller will be unable to track the reference perfectly, increasing the net fuel use; this cost is not factored into the analysis in this paper because it is controller specific and represents an additional, variable cost beyond the determination of the fuel optimal trajectory.

**Figure 2:**
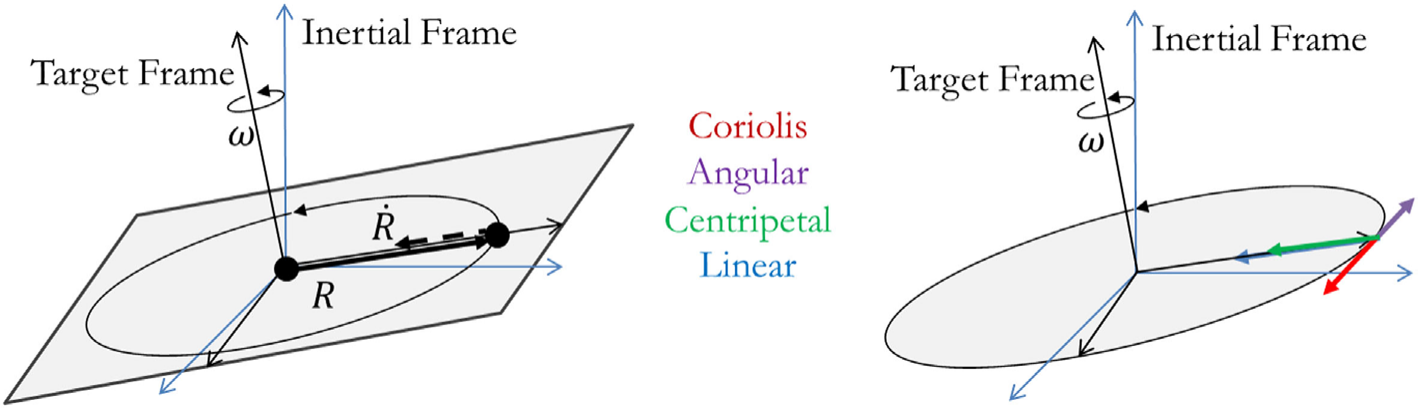
Acceleration component diagram: position and velocity vectors of the approaching chaser are shown on the left, and the four acceleration components are shown on the right.

The accelerated trajectory requires the chaser’s thrusters to generate four types of acceleration. Equation 7 has five parts: the first is the summation of the four acceleration terms (linear, Coriolis, angular, and centripetal) that comprise the total acceleration $\left(\ddot{r}\right)$ seen by the Chaser on its approach. The remaining four parts are the expressions for each of these accelerations. In each equation, the radial distance from the Target’s center of rotation to the Chaser’s center of mass is given as *R* and the angular rate shared by both Chaser and Target is given by ω. In these expressions, the variables are 3 × 1 vectors with components describing the quantity of interest with *R* and ω being described in the Target’s frame. The components are shown graphically in Figure 2. Additionally, these acceleration components do not account for any rotation of the Chaser satellite as it maintains its relative orientation to the Target; these accelerations are assumed to be provided by means other than thruster systems, such as reaction wheels or control moment gyroscopes. Should thrusters be used instead, an additional fuel penalty would be necessary to account for the need to maintain the appropriate docking attitude. Owing to the scale of expected Chaser satellites, such a fuel cost would be small in comparison to the fuel required to move the Chaser throughout its terminal approach profile. This cost is considered to be small because of the ability of the Chaser to change its orientation through rotating its inertia over the same duration as the whole mass must move in the synchronous approach. Consequently, the rotation of the body is less than the translation of the body when over the same duration and while maintaining center-pointing

(7) $$\begin{array}{l} \ddot{r}=a_{\text{linear}}+a_{\text{coriolis}}+a_{\text{angular}}+a_{\text{centripetal}}\\ a_{\text{linear}}(t)=\ddot{R}\\ a_{\text{coriolis}}(t)=2\omega \times \dot{R}\\ a_{\text{angular}}(t)=\dot{\omega }\times R\\ a_{\text{centripetal}}(t)=\omega \times (\omega \times R). \end{array}$$

The computation of these time histories for each acceleration term is performed using a numerical propagator. The initial conditions listed above are set along with the properties of the Target satellite. A sequence of time steps, *dt_i_*, are computed to span the radial distance from *r*_0_ to *r_f_*, with *i* = 1:100. The acceleration components are computed at each time step, with the total acceleration being computed as the norm of the vector sum of all components. In this manner, the total acceleration accounts for potentially opposing acceleration terms, and relies on the Chaser being able to provide the corresponding thrust level in the same direction as the resultant total acceleration vector. The inability to provide this thrust would serve as an inefficiency factor, though the overall computation process would remain unchanged.

The computation of the acceleration terms in Eq. 7 enable the computation of the Δ*V* required to perform the approach maneuver. The integration of each acceleration component reveals the corresponding Δ*V* cost. For example, a Chaser spiraling radially inward at a constant speed to a Target in a flat spin with a constant rotation rate would have no linear acceleration component except for an initial impulse to begin the inward motion, a constant Coriolis component, no angular component, and a shrinking centripetal acceleration component. The largest component for both acceleration and Δ*V* for the majority of the approach is centripetal. The linear Δ*V* component is constant after the initial inward thruster firing, and the angular component is 0 m/s. Furthermore, since the Coriolis acceleration is a constant, the corresponding Δ*V* is linear over the course of the approach. Equation 8 shows how the net Δ*V* is obtained through the integration of the total acceleration. This approach accounts for the orthogonality between the tangential (Coriolis and angular) accelerations and the radial (linear and centripetal) accelerations. Additionally, this method for computing the total Δ*V* prevents double-counting should any acceleration terms act in opposite directions. For example, a radial deceleration would require that the linear acceleration term act in the opposite direction as the centripetal acceleration. In such a situation, the net acceleration vector would be the sum of both terms, so opposing thrusters would not simultaneously fire

(8) $$\Delta V_{\text{total}}(t)=\int_{0}^{t_{f}}(\ddot{r})\mathrm{dt}.$$

This process for computing the acceleration and Δ*V* demands of trajectories is applied for all Targets, including those that are tumbling with varying levels of precession and nutation as dictated by inertia ratios and angular rates. All the Target properties are prescribed at the start of each propagation simulation and are unforced, which is in agreement with the overall goal of this paper corresponding to rigid, naturally tumbling, and uncooperative Targets. The approach in this section for computing the Δ*V* for each trajectory, therefore, enables solvers to determine the optimal time steps between radially spaced waypoints to minimize the total Δ*V* needed. The solver can, therefore, be used to determine fuel optimal trajectories for the Chaser satellite to follow as it approaches the Target.

## Generating the Fuel Optimal Trajectory

In order to make comparisons between full trajectory optimizations and reduced parameterizations, a common method for generating trajectories must be established. This method relies on the prior section’s propagation of the Target’s tumble through the time of docking and the subsequent computation of the Δ*V* cost to the Chaser. Therefore, the following sequence of steps is performed to determine the optimal trajectory for a specified set of boundary conditions and the corresponding reduced parameterization of that optimal trajectory:

1. Find the fuel optimal trajectory.
  - Discretize the terminal approach into a sequence of radial steps from *r_0_* to *r_f_*.
  - Find the time steps *dt_i_* for a fixed *t_f_* for each radial step that result in a minimization of the net required Δ*V*.
2. Fit a parameterized approximation to the fuel optimal trajectory.
  - Try fitting different curve expressions to the fuel optimal radial trajectory.
  - Evaluate fit accuracy vs. the number of required parameters.
3. Compare optimal and parameterized trajectories.
  - Select the reduced parameterization that balances fit accuracy with computational efficiency.
  - For the same boundary conditions across several tumble types, assess the computation time and fuel cost of the reduced parameterizations.

The process in step 1 is performed by finding the optimal time history for the Chaser satellite to travel between 100 radially spaced points. The number of radially spaced points affects the computational complexity of computing the optimal trajectory and the level of discretization of the true optimal trajectory (a continuous path). This analysis uses 100 points to represent the true optimal trajectory, since this number is computationally tractable while affording time steps on the order of no more than a few seconds for expected docking approach durations.

The choice of radially spaced points affects how well the Matlab fmincon solver can identify the optimum sequence of time step lengths. Because the solver must find the value for an arbitrary number of time steps, each time step represents a variable whose value must be identified, a process that can become computationally inefficient rapidly. The number of time steps is taken as 100 for this paper to balance the computational requirement and the accuracy of the resulting optimal trajectory, since a forward propagation using Euler’s equations is used to determine the states of the Chaser and Target over time. Spacing the radial points was performed *via* linear and logarithmic means. In the linear case, the radial waypoints maintained a constant separation from one another over the full range of initial radius to final radius. Consequently, the linear spacing tasked the solver with finding the time steps between points that were uniformly distributed over the desired radial span. The logarithmic spacing, however, generated a radial distribution with points spaced between logarithmic decades, thereby increasing the concentration of points nearer the Target. The solution that is provided by the optimization solver is dependent on initial values. From the scenarios tested in this paper, the results were unchanged despite changing the initial guess to linear or exponentially decreasing radial distances.

**Figure 3:**
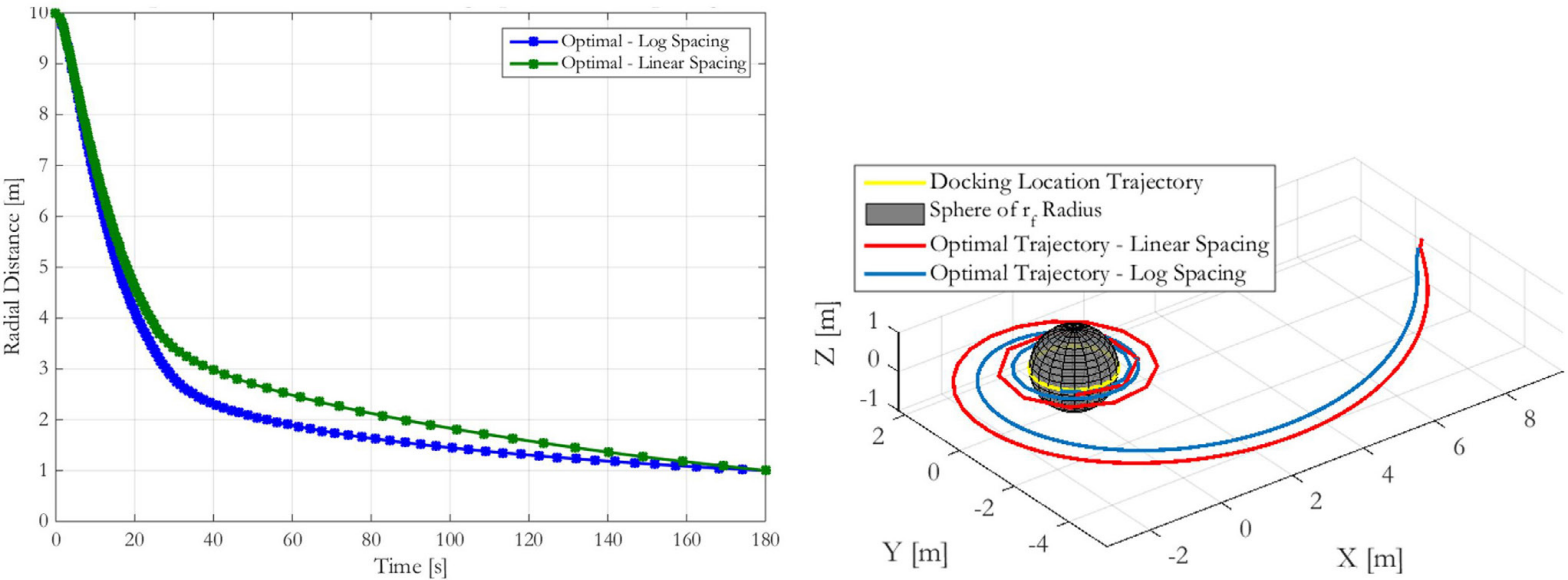
Radial approach comparison between linear and logarithmic optimal radial spacing (left) and comparison of inertial frame trajectory of linear and logarithmic optimal radial spacing (right).

Because the final radius is smaller than the initial radius, the additional points near the Target provided by the logarithmic spacing over the linear spacing method help to provide a smoother trajectory with more uniform *dt* time step sizes. This result may be illustrated by an example where a Chaser approaches from 10 m a Target of 1 m final radius rotating in a flat spin at 5 deg/s. The optimal trajectory was found using both linear and logarithmic radial point spacing, with each being shown in the left plot of Figure 3. While both trajectories carry the Chaser satellite from its initial point to the docking location, the logarithmic spacing approaches the Target quicker over the course of the prescribed 180 s maneuver. Additionally, though both the linear and logarithmic spacing time steps averaged to 1.8 s (180 s trajectory broken into 100 time steps), the SD of the time steps is 1.7 s for the logarithmic spacing and 2.8 s for the linear spacing. The logarithmic spacing’s smaller variability in its time steps enables it to provide improved Δ*V* performance over the linear case, with the logarithmic case being able to provide improved fuel use by up to approximately 15% over the linear case. The resulting trajectory is smoother because of the more uniform time steps across less uniform radial points.

**Figure 4:**
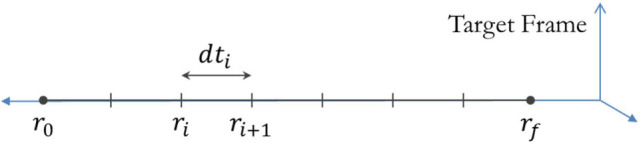
Schematic showing that the optimization process occurs over the time taken between radial steps along the synchronous terminal approach.

This behavior may also be observed by plotting the Chaser’s approach in both cases, as shown in the right plot of Figure 4. The logarithmic spacing provides a smoother approach particularly nearer the Target, where the linearly spaced points with corresponding long time steps create visible segments of the approach. The resulting acceleration spikes at these locations require additional Δ*V*, thereby increasing the net fuel cost for these trajectories that is higher than the fuel cost for the logarithmic spacing. Consequently, the analysis presented in this paper centers around the use of logarithmically spaced points for determining fuel optimal trajectories.

While this figure focuses on a single example, the results may be generalized. In particular, the poor performance near the Target exhibited for small radii (near the Target) by the linearly spaced points becomes exacerbated for larger rotation rates or smaller radii, i.e., the conditions which create large accelerations as the Chaser moves along comparatively long line segments. A limit case occurs when the time step takes the Chaser between radial points which, on account of the rotation of the Target, may be connected by a line segment which intersects the Target spacecraft. Solving for optimal *dt* steps between logarithmically spaced radial points, therefore, mitigates this effect without the need to increase the number of time steps (and the associated computational cost) and enables trajectory generation for range of tumble types.

In step 1a, the points are spaced logarithmically such that a higher number of points are placed nearer the Target. The optimization process centers on the determination of a large number of values, in this case time steps between radii, as determined by numerical propagation of the Chaser’s state. This process is computationally expensive, since each of the 100 time steps must be optimized. Computing the fuel minimum trajectory, therefore, requires the computation of the 100 variables that minimize the fuel consumption of the Chaser, a task that requires nonlinear constrained optimization. Matlab’s fmincon function is used to perform the optimization with the options set to allow 100,000 maximum function evaluations, as well as a “TolFun,” “TolX,” and “TolCon” of 1 × 10^−8^. The initial guess that is provided to the solver is an evenly spaced set of time points (constant *dt* steps) from *t* = 0 to *t* = *t_f_*. The initial condition for this optimization is shown by the schematic in Figure 4. The optimization function propagates the Target and Chaser over the course of the time history along the radial distance from *r_0_* to *r_f_*, computing the Δ*V* for each functional evaluation of a set of time step vectors. When a solution that meets the fmincon constraints for finding a minimum cost is found, the resulting Δ*V* is reported as the minimum fuel required to perform the approach. The resulting time sequence of *dt_i_* represents the optimal time history for the initially provided radial profile. Together, the optimal trajectory can be plotted, completing step 1b.

## Parameterizing the Fuel Optimal Trajectory

To avoid a computationally expensive full optimization, thereby enabling the Chaser satellite to update its trajectory in real-time while approaching the Target, a smaller number of parameters is necessary to describe the shape of the trajectory. This reduced parameterization decreases the number of free variables for an optimization, but yields a more fuel-costly trajectory. The task for mission planners is to trade the computational complexity and processing time of generating the trajectory against the fuel cost of the trajectory. In seeking an efficient balance between the two metrics, it is possible to study different parameterizations of an optimal trajectory and determine their comparative ability to approximate a fuel optimal radial path in step 2.

### Identifying Reduced Parameterization Expression

To determine the appropriate parameterization of the optimal trajectories, there are two aspects to consider: the fit approximation type and the number of parameters used in the fit. Fitting with sums of terms (like polynomials and exponentials) or existing functions (like the generalized logistic function) may be performed. These expressions are described in Table 1 and are, therefore, the fit functions used in step 2a. In this table, the expressions include up to six optimization parameters. Since the expressions have different numbers of parameters per term, comparisons must be made based on the number of parameters, not the number of terms. The upper limit of the summations corresponds to this difference in total number of fit terms.

**Table 1:** Parameterization expressions for optimal trajectory fitting.

Fit type	Expression requiring six parameters
Polynomial	$r(t)=\sum_{i=0}^{5}b_{i}t^{i}$
Exponential	$r(t)=\sum_{i=1}^{3}b_{i}{\text{e}}^{-c_{i}t}$
Generalized logistic function	$r(t)=L_{1}+\frac{L_{2}-L_{1}}{{\left(L_{3}+L_{4}{\text{e}}^{-L_{5}t}\right)}^{\frac{1}{L_{6}}}}$

Importantly, this set of expressions inherently provides several properties. The polynomial terms each have a factor of time to a power, with that power growing with the number of added terms. As a result, the coefficients of these time factors have a greater impact on the net fit than those of lower powers of time, especially for longer duration maneuvers. This negative effect is offset by the ability for this parameterization to continue improving its approximation with higher numbers of terms since each new term can improve the fit for particular sections of the trajectory, such as during the later stages of an approach. The exponential fit’s expression, however, is comprised of identical, two-parameter terms. Consequently, the terms allow for improved fits with increasing numbers of terms, but must add two parameters at a time. Unfortunately, the identical form of each term can engender numerical ill conditioning with larger numbers of terms. This ill conditioning arises because each term has the same impact as the rest when computing the optimal set of parameters. As a result, it is possible for the leading parameters to become several orders of magnitude larger than the parameters in the exponentials. In this way, the polynomial fits avoid the term cancelation issue seen by the exponential fits, but instead exhibit large differences in the comparative contributions of the different terms over the duration of the docking maneuver. The generalized logistic function has several shaping parameters and cannot be expanded with additional terms; it is, therefore, able to be modified only by the choice of its six parameters. Termination criteria in the optimization can, therefore, have a large effect on the final parameter values.

The fitting process in step 2b is performed *via* a least squares approach. The optimal trajectory is first determined, providing the optimal time history associated with the initially chosen radial spacing. In this analysis, the fit functions are hardcoded to accept input vectors containing the parameters that define the order of the expression. The least squares fitting process again uses the Matlab fmincon function with the same set of user-defined options, but with the function for minimization, *J*, being:

(9) $$J=\frac{1}{n}\sum_{i=1}^{n}\left|r_{\text{opt},i}-r_{\text{fit},i}\right|.$$

In this equation, *r*_opt_ is determined by the aforementioned optimization process, while the *r*_fit_ radial distribution is determined for the *i*th iteration of the fitting optimization. Both *r_0_* and *r_f_* must be matched and are, therefore, treated as equality constraints. The initial guess for fmincon is taken to be that which provides the one-term fit that matches *r_0_* or a plausible trajectory. Therefore, the initial guess in the polynomial case is *b*_1_ = *r_0_*, the initial guess for the exponential case is *b*_1_ = *r_0_, c_1_* = 0, and the initial guess for the generalized logistic function is *L*_1_ = 10, *L*_2_ = *L*_3_ = *L*_4_ = 1, *L*_5_ = *L*_6_ = 0.1. The higher order expressions use the solution from the lower order fits as their starting points; this approach necessitates that *n* fits must be performed to determine the best fit of an *n*-term expression. There is the potential to find local minima using this process. To minimize the likelihood of this occurrence, the fmincon options were set to allow large numbers of iterations with tight constraints. Furthermore, the bounds on the possible parameter values are kept open to give the fmincon solver flexibility in its searching. These options are set prior to calling the optimizer, and the output of the optimizer is saved for the forthcoming analysis. Separate functions are written for the cost function, nonlinear constraint function, and the analysis function. Additional details about the optimization process may be found in Sternberg (2017).

**Figure 5:**
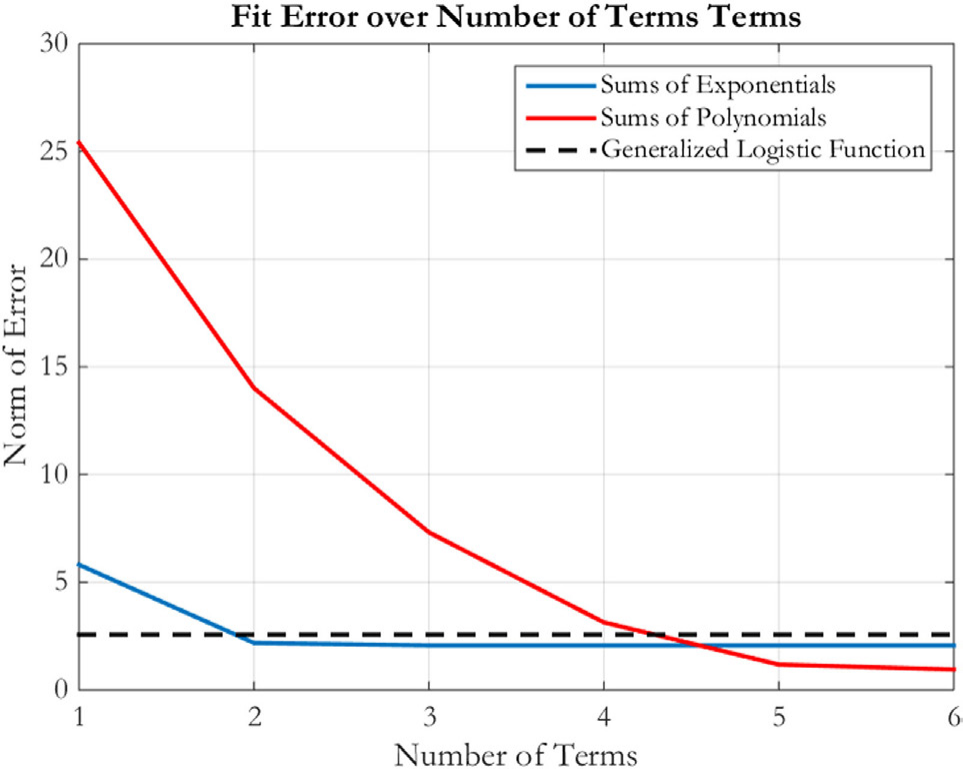
Fit error over number of terms for sums of exponentials, sums of polynomials, and the generalized logistic function fits.

The fit accuracy must be assessed as a function of the number of parameters in step 2b. The number of parameters is a measure of the optimization complexity, while the accuracy is the cost of the parameterization. Figure 5 shows a comparison of the fit error from Eq. 9 as the one-norm of the distance between three fit functions and the optimal trajectory for a flat spin from 10 to 1 m in 2 min while rotating at 5 deg/s. The horizontal line in Figure 5 shows the error level that is found when fitting the generalized logistic function; its error level provides a threshold where points below the line offer improved fits over the generalized logistic function. The error curves for both sums of exponentials and sums of polynomials cross this threshold, but at different locations. Sums of exponentials provide improved fitting performance than generalized logistic functions with two terms (four parameters), and sums of polynomials provide improved fitting performance with five terms (five parameters). Importantly, there is a marked decrease in the fit error for polynomial and exponential fit methods at four parameters, where thereafter little fit improvement is acquired with additional parameters. These expressions match fit error with five parameters. For larger numbers of exponential terms, the ill conditioning described above prevents any significant improvement in the fit quality.

**Figure 6:**
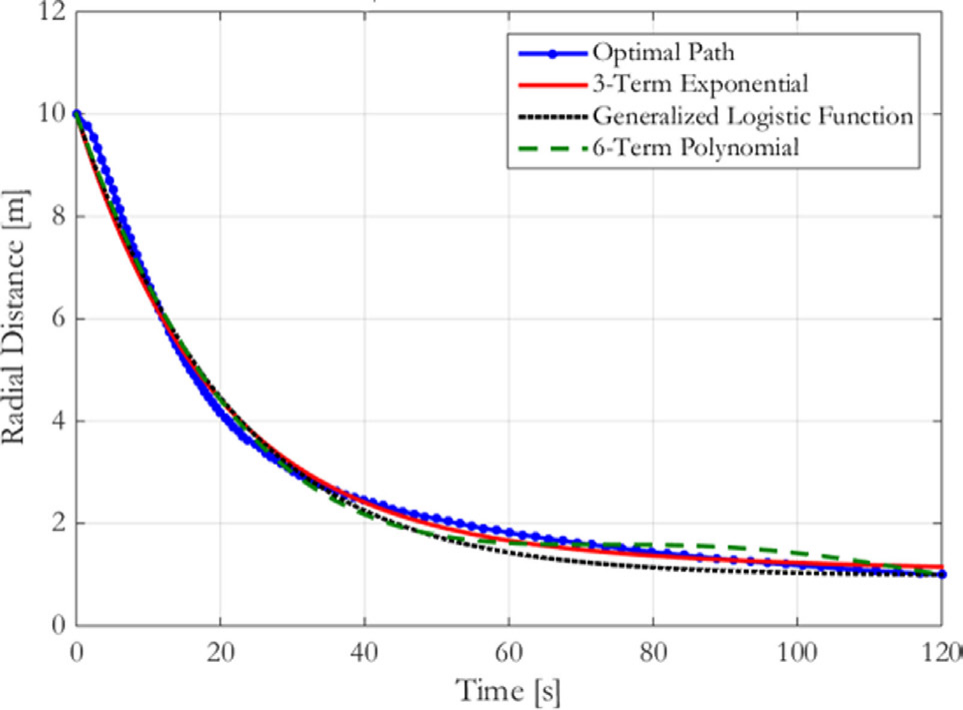
Radial distance for flat spin from 10 to 1 m at 5 deg/s: comparing fits with six parameters each.

Figure 6 shows the radial history for the three fits using six parameters each. The generalized logistic function undershoots the optimal trajectory for approximately the second half of the spiral. Doing so also increases the collision risk with the Target by operating in close proximity for an extended duration, while requiring additional fuel to move quickly inward toward the target. The exponential fit provides a good approximation for the optimal trajectory, primarily because it does not exhibit the oscillations found in the polynomial fit during the second half of the approach. These oscillations are damped with increased numbers of terms, improving the fit beyond the exponential case. With six parameters, the oscillations keep the polynomial fit around the optimal trajectory instead of remaining further from the optimal trajectory. Nevertheless, both perform similarly to one another at this number of parameters. Because the goal of the designer is to trade computation cost with the performance of the approximation of the optimal trajectory, this paper continues the analysis with the sums of exponentials, since this method enables fits with low numbers of parameters to outperform polynomial fits while being nearly equal in the ability to approximate the optimal trajectory with larger numbers of terms. Therefore, step 3a has resulted in sum of exponential parameterizations being selected as the reduced parameterization method.

This analysis in this subsection demonstrates that a low-order parameterization effectively matches the high-order optimal trajectory in this example. It is, therefore, necessary to assess how well the parameterization can reduce Δ*V*, while remaining low order for other tumble scenarios. While the fit error indicates that an effective trade between computational complexity and closeness of the fit approximation occurs at four parameters, it is possible to compare the fuel use for a series of tumbles to assess the exponential fit’s ability to minimize fuel use while also minimizing the number of required parameters to be included in the optimization process.

### Generating the Fuel-Optimal Parameterized Trajectory

The process for finding the fuel optimal exponential trajectory is similar to that of finding the optimal trajectory. The optimization uses Matlab’s GlobalSearch tools along with the fmincon solver. Instead of solving for the time step sequence that results in the optimal trajectory, the optimizer searches for the parameters of the fit function. A time step vector is created as a linearly spaced vector from *t* = 0 to *t* = *t_f_*. This method of stepping the propagation over the duration of the approach is complementary to the approach used to generate the optimal trajectory. Instead of a logarithmically spaced set of radial points, a linearly spaced set of time points are selected for the optimization to determine the best radial approach profile. The switch in optimization methods enables the radial approach profile to be defined by the set of optimization variables. The radial distribution is computed from the trajectory parameters: the equation defining the sum of exponentials trajectory is used with the corresponding number of parameters. A nonlinear equality constraint function, shown in Eq. 10, is used to match the starting and final radii of the fit radial distribution

(10) $$c_{\text{eq}}=\left[\begin{array}{c} r(t_{f})-r_{f}\\ r(t_{0})-r_{0} \end{array}\right].$$

The same optimization options for fmincon are used again, and the optimal parameters are output for the desired number of terms. The initial guess for fmincon is a parameterized trajectory that meets the boundary conditions. For this paper, the selected initial guess is the two-term exponential fit to the optimal trajectory. Because this GlobalSearch process optimizes the parameters for a fit expression, the computational complexity is lower than that of an optimization of the full trajectory. By optimizing up to six-term exponentials, the number of variables to be optimized drops by an order of magnitude, saving computation time for the Chaser.

**Figure 7:**
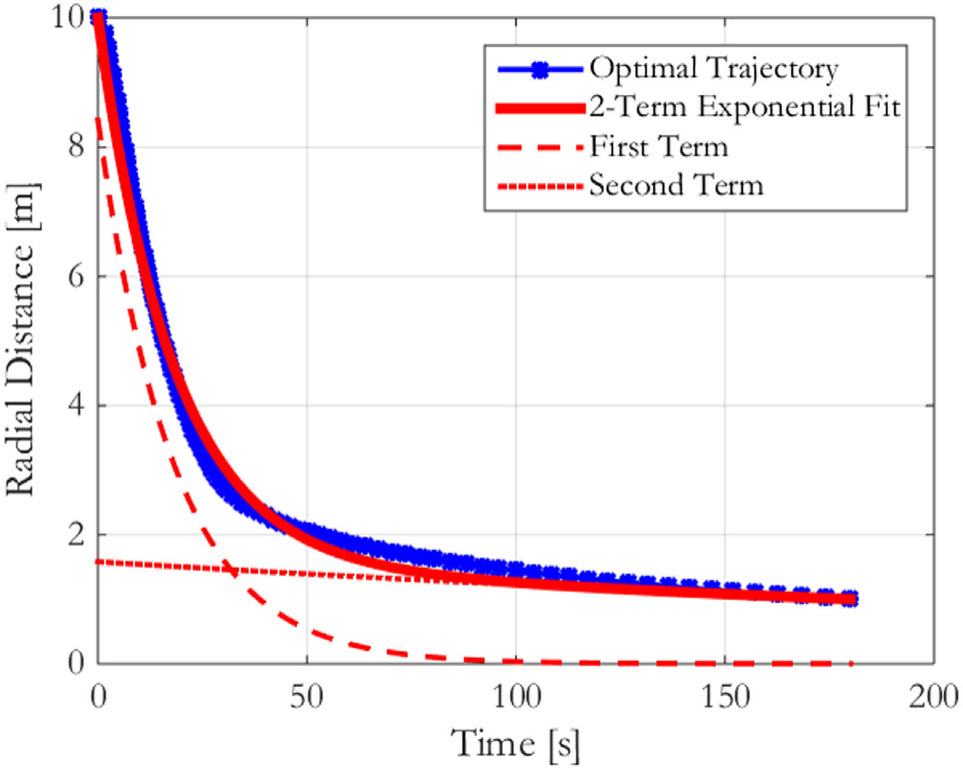
Comparison between the optimal trajectory and the optimal two-term exponential trajectory with its two component terms for a flat spin at 5 deg/s from 10 to 1 m in 180 s.

The comparison between the optimal trajectory as determined by finding the optimal time spacing between radial points and the optimal trajectory as determined by finding the optimal two-term exponential parameters is shown in Figure 7. The optimal trajectory is approximated by the sum of two exponential terms in which each approximate a different portion of the optimal trajectory. The first term approximates the slope of the optimal trajectory during the initial steep descent when the Chaser satellite moves rapidly toward the Chaser as a means of avoiding excessive centripetal acceleration fuel losses. The second term approximates the shallower slope during the time when the Chaser moves slowly toward the Target to ensure docking occurs at the specified time (*t_f_*). Therefore, the dynamics of the approach provide an additional means of selecting the number of terms with which to match the optimal trajectory: the optimal trajectory’s two regimes can be approximated by two decaying exponential terms.

The fuel minimizing two-term exponential trajectory, an approximation to the optimal trajectory, requires that substantially less computation with only a slight increase in fuel use. Table 2 shows that for this example, the fuel cost is only 10% above the optimal trajectory’s fuel requirement, while the computation time has been reduced by a factor of four. Although the optimizations both required the use of Matlab’s fmincon function with the same effective objective function (to minimize the fuel cost of a trajectory), the two optimizations show that the computation cost does not scale linearly with the number of parameters being optimized. It is expected that the optimization over parameters that individually have greater effects on the cost function in computing the optimal two-term exponential trajectory is a key contributor to its computation cost.

**Table 2:** Δ*V* and computation time cost comparison between the optimal and two-term exponential trajectories.

Example	Optimal	Two-term exponential
Δ*V* Percent	100	109
Computation time percent	100	23

**Figure 8:**
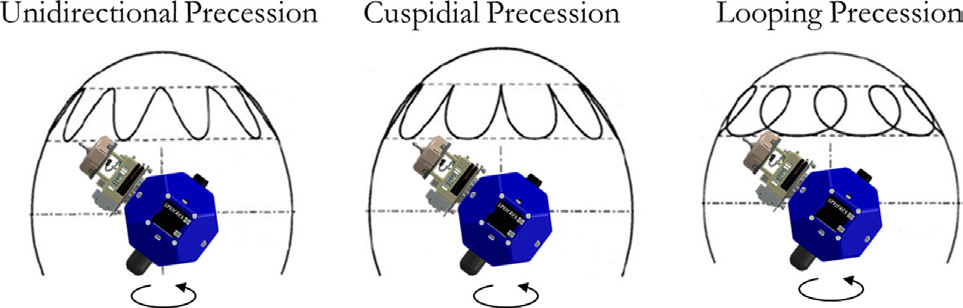
Diagrams of precession types for tumbling Target satellites.

**Figure 9:**
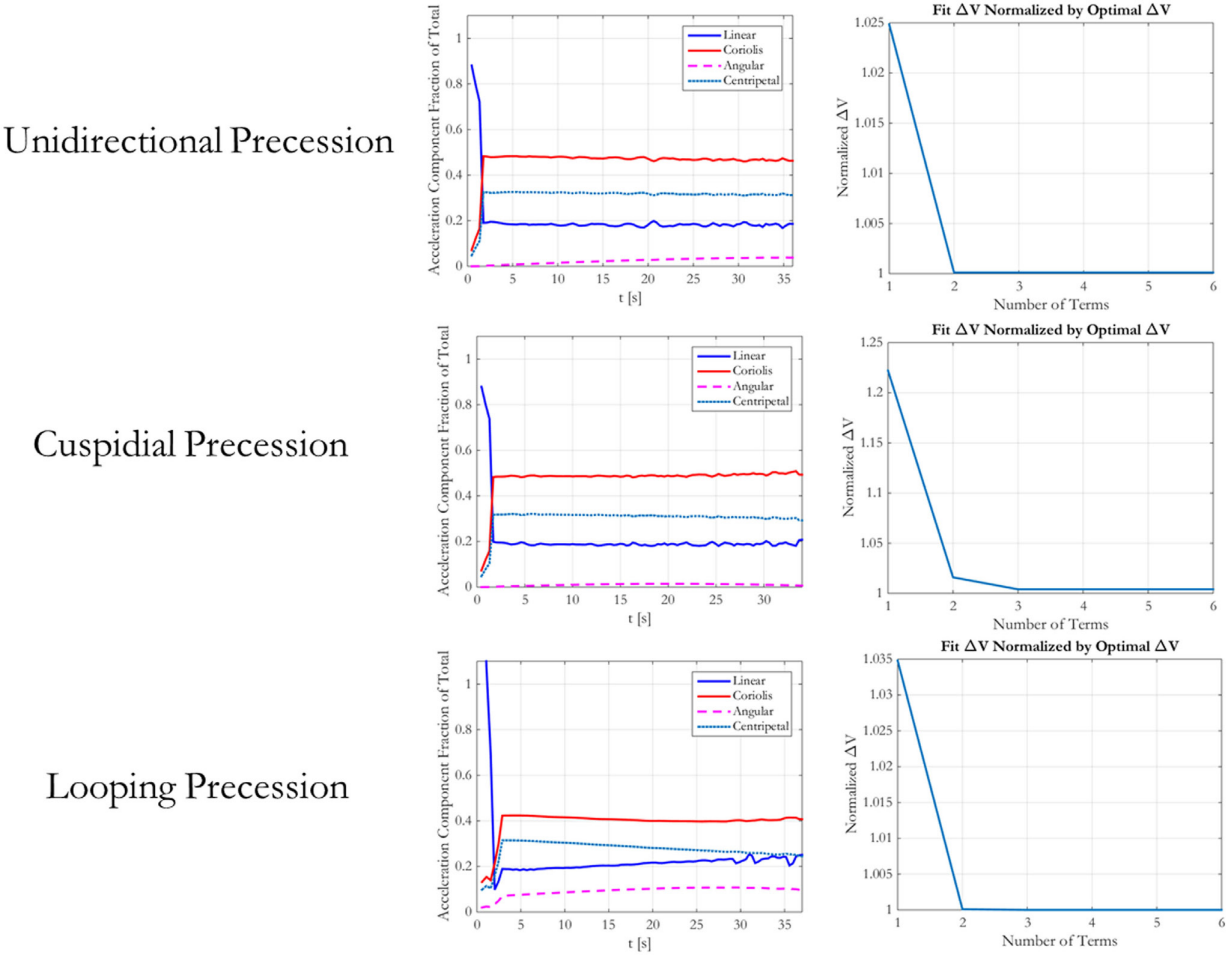
Fitting increasing number of exponential terms to various tumble types.

While the above analysis assessed the Δ*V* required by the best *n*-term exponentials for a flat spin case, it is also important to assess the fuel required by these trajectories for multiple types of tumbles. Figure 8 shows a notional trace of a docking port motion for three types of Target docking port motions. These motions result from a Target’s spin with different combinations of spin vectors and inertia ratios. The cuspidial precession case is a limit case between unidirectional and looping precessions. Because the synchronicity constraint is determined by the motion of the Target’s docking axis, the path traced in these diagrams represents the changing path that the Chaser must also follow. Figure 9 shows a matrix with each row corresponding to a type of precession that matches the diagrams in Figure 8. The left column states the type of precession. The central column shows the acceleration components for the optimal trajectories as fractions of the total acceleration at each time step. The right column shows the required Δ*V* for that trajectory normalized by the Δ*V* required by the optimal trajectory for the corresponding set of boundary conditions. These figures, therefore, also span the types of expected motions seen by on-orbit Targets, and they also provide additional evidence that two-term exponentials provide a balance between the computational complexity of finding the fuel optimal parameterization and the corresponding required Δ*V*. The rotation types presented in the figure include the three different types of precession that may be seen, with cuspidial representing the limit case between unidirectional and looping. Other cases, such as non-tumbling or axial rotations, are combinations of one or more of these three cases with different amplitudes of motion or axes of rotation. The worst case in this figure is that of cuspidial precession, where two- or three-term exponentials may be selected to provide fuel savings while requiring less computational time. The reduced parameterization, therefore, may be taken to be that of two-term exponentials, parameterized as $r(t)=b_{1}e^{-c_{1}t}+b_{2}{\text{e}}^{-c_{2}t}$, because they provide the bulk of the fuel savings over the range of potential tumbles.

Table 3 shows a comparison of the required time to compute the optimal trajectory by the two methods of optimizing for 100 time steps or for the four parameters of a two-term exponential. The optimizations were run for 243 total trials as determined by a full factorial of the parameters included in the table. The resulting data shows that the optimization across all of the cases is approximately three times more computationally expensive on average for determining the optimal 100 point trajectory, showing the benefit for employing the reduced parameterization for determining the fuel minimizing trajectory.

**Table 3:** Comparison of computation times required for both 100 point optimal and two-term exponential trajectories for specified parameters for 243 simulated approaches.

Parameters for full factorial of 243 runs	*r_0_* (m) *r_f_* (m) ω (deg/s) *J* *t_f_* (s)	[5 10 20] [0.5 0.75 1] [5/|[0 0 1]| [0 0 1]^T^] [5/|[1 1 1]| [1 1 1]^T^] [5/|[0 0.05 1]| [0 0.05 1] [1 1 1]^T^ [1.2 1 1]^T^ [2 2 1]^T^ [30 60 120]	

		100 point optimal	Two-term exp. optimal
Computation time statistics (s)	Max	649.8287	116.3459
	Min	66.0904	57.7495
	Mean	238.2191	77.5673
	Median	191.4989	76.2070
	SD	135.1957	10.2613

This section has found fuel minimizing trajectories and a parameterization method to balance the computational complexity of determining a terminal approach trajectory with the fuel cost associated with the parameterization. This process was studied, however, for set values of *t_f_*, providing a means of determining the fuel optimal trajectory by sweeping through *t_f_* values.

## Conclusion

This paper has addressed the need for the rapid generation of fuel minimizing approach trajectories to Targets that may be tumbling. Computational efficiency and fuel optimality have been assessed over several approximation functions with varying numbers of parameters. This optimal trajectory was generated through the optimization of 100 radially spaced points along the docking axis. The approximations of this optimal are reduced parameterizations and are, therefore, able to afford computational efficiency by decreasing the number of optimization variables. The two-term exponential parameterization was shown to closely approximate the optimal trajectory for a representative set of Target tumbles, demonstrating the generality of this reduced parameterization to multiple types of future mission applications.

## Author Contributions

Substantial contributions or design of the work; or the acquisition, analysis, or interpretation of the data for the work: DS and DM. Drafting the work: DS. Revising it critically for important intellectual content: DS and DM. Final approval of the version: DM.

## Conflict of Interest Statement

The authors declare that the research was conducted in the absence of any commercial or financial relationships that could be construed as a potential conflict of interest. The reviewer, TG, and handling Editor declared their shared affiliation.
